# An interpretable credit risk assessment model with boundary sample identification

**DOI:** 10.7717/peerj-cs.2988

**Published:** 2025-06-30

**Authors:** Runchi Zhang, Iris Li, Zhiyuan Ding

**Affiliations:** 1 School of Economics, Nanjing University of Posts and Telecommunications, Nanjing, Jiangsu, China; 2 Courant Institute of Mathematical Sciences, New York University, New York, New York, United States; 3 Social Sciences, Franklin and Marshall College, Lancaster City, Pennsylvania, United States

**Keywords:** Credit risk assessment, Interpretability, Boundary samples, Noise samples

## Abstract

**Background:**

Interpretability is a key requirement for ensuring that credit risk assessment models are trustworthy and compliant with regulatory standards. Simultaneously, effectively distinguishing between noise samples and boundary samples is crucial for improving the accuracy of credit risk predictions.

**Methods:**

This article introduces a novel credit risk assessment model, Interpretable Credit Risk Assessment Model with Identifying Boundary Samples (IAIBS). The model begins with a logistic regression sub-model that offers strong self-interpretable features. For samples that are not correctly classified, the Attribute Recognition and Perception based on the Distribution of neighboring sample features (ARPD) algorithm is applied to filter out noisy samples and identify boundary samples. A deep learning sub-model is then trained to deeply learn the risk features of these boundary samples. Finally, representative features of all samples are extracted using agglomerative clustering, and the most suitable sub-model is selected for prediction based on the similarity between each sample and the cluster centers.

**Results:**

Experimental results on four public datasets demonstrate that the IAIBS model significantly outperforms 11 baseline models, as confirmed by the Nemenyi test. The model achieved area under the curve (AUC) scores of 89.17, 79.86, 97.48, and 66.03 on the PCL, FICO, CCF, and VL datasets, respectively. With appropriate parameter tuning, the IAIBS model maintains strong generalization ability, and each module contributes positively to overall performance. Additionally, the IAIBS model effectively interprets key predictors and prediction outcomes.

## Introduction

Credit risk assessment in the lending business refers to the process by which credit institutions predict the borrower’s future ability and willingness to repay debts, by analyzing factors such as the borrower’s financial status, credit history and market environment. The implementation of effective credit risk assessment not only helps to reduce the risk of default, protect the interests of credit institutions and reduce credit losses, but also helps to improve the stability of the financial system, prevent and resolve systemic financial risks (Wang, Li & Zhang, 2025). In the digital economy era, as the scale of credit business continues to grow, more and more credit institutions are trying to develop quantitative assessment models to implement credit risk assessment (Yang & Masron, 2024; Lin et al., 2025). Compared with traditional manual assessment methods, the application of credit risk assessment models has shown significant advantages in terms of data processing ability, prediction accuracy, assessment efficiency, personalization and assessment objectivity (Zhang & Yu, 2024).

Early credit risk assessment models were mostly built using statistical methods, representative achievements include the linear scorecard model, the logistic regression model, the naive Bayes model and the discriminant analysis model. These models are simple in structure, with their computational process and results easier for users to understand (Chen, Ribeiro & Chen, 2016). However, in large-scale sample environments, these models are often difficult to identify the multiple heterogeneous risk features contained in the dataset, so the performance is inferior to that of machine learning assessment models (Zhou et al., 2021; Zhang & Yu, 2024). Machine learning assessment models have significant advantages in dealing with high-dimensional and non-linear problems (Baser, Koc & Selcuk-Kestel, 2023), representative achievements include decision trees, support vector machines, artificial neural networks, *etc*., (Luo, Yan & Tian, 2020; Xie et al., 2021; Zhao & Li, 2022). Some researchers have also attempted to build ensemble or hybrid credit risk assessment models and achieved better assessment results, representative models include random forest, AdaBoost, GBDT, XGBoost, lightGBM, *etc*., (Li et al., 2021; Yao et al., 2022; Abedin et al., 2023; Schetakis et al., 2024). Some cutting-edge research has also attempted to build more complex assessment models based on advanced technologies such as deep neural networks, convolutional neural networks, and reinforcement learning. Representative results include Lappas & Yannacopoulos (2021), Mahbobi, Kimiagari & Vasudevan (2023), Meng, Sun & Shi (2024), Wang, Li & Zhang (2025), Xu & Zhang (2025), *etc*.

Although machine learning assessment models show superior performance, they are often considered as “black boxes” due to their large number of parameters and complex structures (Rudin et al., 2022). In recent years, regulators in major economies around the world have introduced a number of requirements for the interpretability of credit risk assessment models. For example, the EU’s General Data Protection Regulation (GDPR) requires that financial institutions’ automated decision-making processes to include disclosure of the underlying logic, and the EU’s Financial Stability Board (FSB) also emphasizes the importance of the interpretability of artificial intelligence and machine learning methods. The “Model Risk Management Guidelines” (SR 11–7) issued by the Federal Reserve of USA emphasizes that risk management models must be interpretable. The “Evaluation Standards for Financial Applications of Artificial Intelligence Algorithms” issued by the People’s Bank of China also indicated that interpretability is an important basis for assessing whether a model is suitable for Chinese financial institutions. Overall, the importance of interpretability in credit risk assessment is becoming increasingly prominent (Chang et al., 2024). Users and regulators need to understand model’s decision logic in order to gain confidence in the model’s predictive results. In recent years, researchers have proposed various methods to improve model interpretability, such as the Local Interpretable Model-Agnostic Explanations (LIME) algorithm and SHapley Additive exPlanations (SHAP) algorithm (Ribeiro, Singh & Guestrin, 2016; Lundberg et al., 2020). There are also studies that combine multiple sub-models with self-interpretable features to improve accuracy and interpretability (Talaat et al., 2024; Kraus et al., 2024; Nazemi & Fabozzi, 2024).

In addition, in large-scale sample environments, some studies have found that identifying noise samples and boundary samples is of great importance for improving the performance of various models (Yao et al., 2022). Noise samples often interfere with the model’s formation of robust risk feature identification rules, while boundary samples are generally located on the distribution boundary between samples with different labels and are key samples that affect the model’s risk feature identification ability. Many cutting-edge studies have attempted to incorporate the process of noise sample removal and boundary sample identification into the construction of credit risk assessment models, found that it can significantly improve the model’s assessment accuracy (Tian, Yong & Luo, 2018; Yotsawat, Wattuya & Srivihok, 2021; Pang, Wang & Li, 2024; Rao et al., 2024). However, most of these algorithms are based on machine learning models with complex structures and still lack interpretability.

In summary, in the large-scale sample environment, more accurate identifying noise samples and boundary samples, while maintaining the interpretability of credit risk assessment models, is crucial to improve the accuracy of credit risk assessment and meet the requirements of regulators regarding the interpretability of models. In this regard, this article designs the IAIBS model. It first uses a logistic regression sub-model with good self-interpretable features to identify the risk features of most samples. For misclassified samples, the proposed ARPD algorithm is applied to determine whether they are boundary samples or noise samples, and then the deep learning sub-model is trained based on the boundary samples to fully identify their risk features. Finally, agglomerative clustering is performed on the subsets of samples correctly identified by the logistic regression sub-model and the subsets of boundary samples, and the most appropriate sub-model for credit risk assessment is selected based on the spatial similarity between each sample to be evaluated and the center of each cluster.

Compared to existing similar research achievements, the innovations of this article are: (1) A novel credit risk assessment model that is both accurate and interpretable is constructed, namely the IAIBS model. The IAIBS model designs differentiated interpretable sub-models for different classes of samples, which effectively improves the accuracy. (2) A novel algorithm for identifying sample attributes is designed, namely the ARPD algorithm. It determines whether each sample is a noise sample or a boundary sample, based on the spatial neighboring sample features of each sample and applies statistical inference methods. The empirical research results show that it can effectively improve the accuracy of credit risk assessment.

## Materials and Methods

The IAIBS model first constructs a logistic regression sub-model with good self-interpretable features on the initial training dataset $D$, the basic structure of the logistic regression model can be summarized as shown in Eq. (1):

(1) $$\matrix{ {P({Y_i} = 1|{X_i}) = \displaystyle{1 \over {1 + {e^{ - \left( {{\beta _0} + \mathop \sum \nolimits_{a = 1}^K {\beta _a} \times x_a^i} \right)}}}}} \cr }$$

where ${X_i}$ represents the sample vector $i$, ${Y_i} = 1$ marks ${X_i}$ as a default sample and ${Y_i} = 0$ marks ${X_i}$ as a non-default sample, $P({Y_i} = 1|{X_i})$ represents the default probability of ${X_i}$ estimated by the model. ${\beta _0}$ and ${\beta _a}$ are parameters to be fitted, $x_a^i$ is the value of dimension ${\rm a}$ of ${X_i}$, $K$ is the total number of dimensions, $e$ is the natural base.

Assume there are $m$ samples that are misclassified by the logistic regression model (that is, the actual non-default samples are predicted as default ones, or the actual default samples are predicted as non-default ones). Obviously, these $m$ samples are similar to samples that adjacent to them in the sample space and have different labels, so that the logistic regression model cannot fully identify the feature differences between these two classes of samples. For each sample ${X_i}\left( {i \in \left\{ {1,2, \cdots ,m} \right\}} \right)$ of these $m$ samples, IAIBS model believes that it is either a sample that is close to the distribution boundary, that is, a boundary sample; or it is a sample that has a different label with other neighboring samples in the training set, due to errors in the data collection or annotation process, or due to the rare feature distribution itself, making it difficult to be classified correctly by the model, that is, a noise sample. The IAIBS model needs to further eliminate noise samples and identify the risk features contained in the boundary samples.

**Figure 1:**
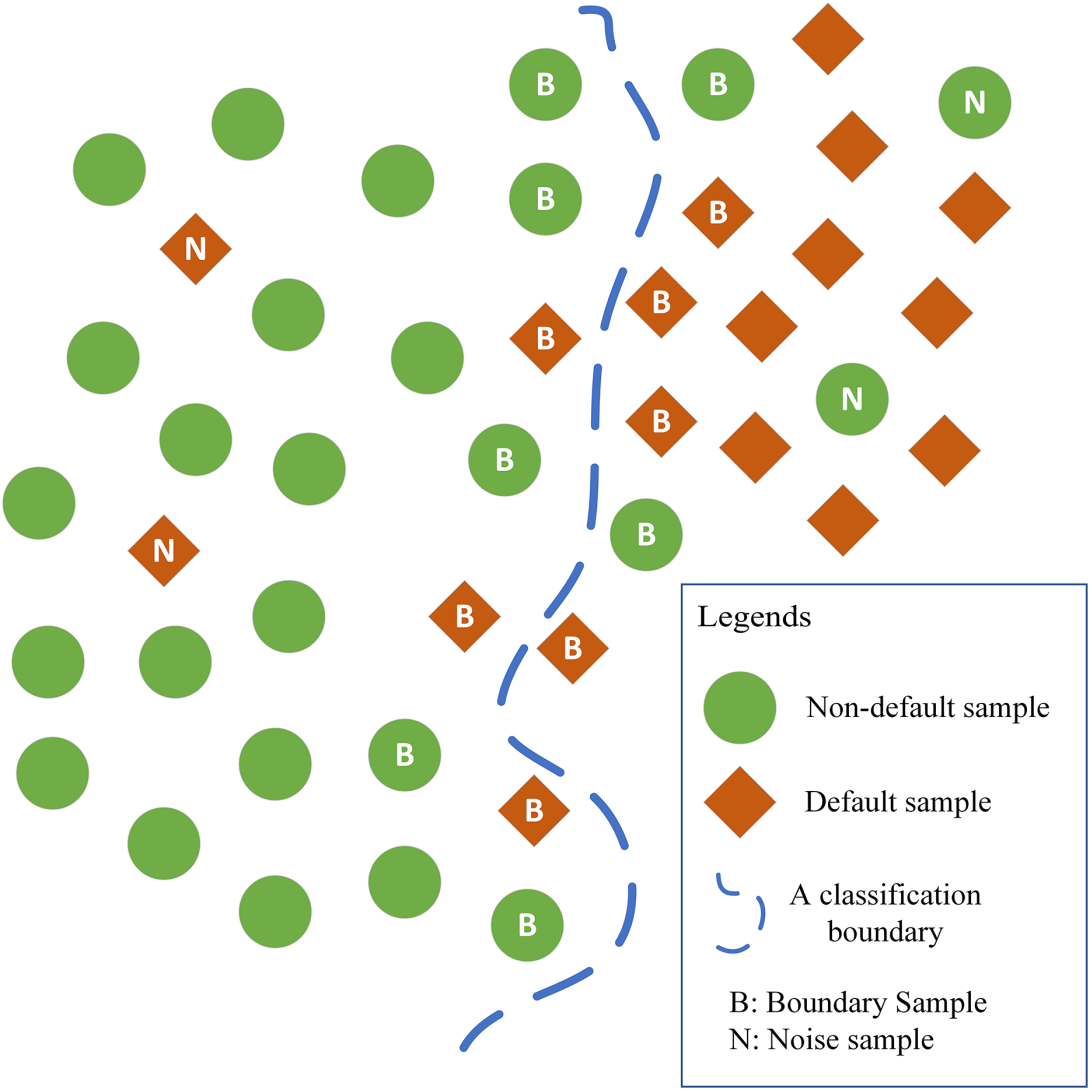
Distribution of neighboring samples of noise samples and boundary samples.

In this regard, the ARPD algorithm is designed. The core idea of the ARPD algorithm is that in the sample space, although the boundary samples and noise samples themselves have certain similarities with their neighboring samples, the distribution of the labels of these neighboring samples is always different. Among the neighboring samples of the boundary samples, two classes of samples are almost equally distributed, making it difficult for the logistic regression model to identify the non-linear classification boundary. Among the neighboring samples of the noise samples, there are usually fewer samples with the same labels and more samples with different labels. The corresponding distribution features can be summarized as shown in Fig. 1.

Thus, the attributes of misclassified samples can be identified by observing the distribution of their neighboring samples. Specifically, the ARPD algorithm first assumes that the number of samples with the same label in the neighboring area of a boundary sample follows the Bernoulli distribution as $B\left( {n,p} \right)$, where $n$ is the number of samples of the nearest neighbor, $p$ is the probability of draw out a sample with the same label when sampling once. Under the law of large numbers, to identify each sample ${X_i}\left( {i \in \left\{ {1,2, \cdots ,m} \right\}} \right)$ is a boundary sample or a noise sample, we can observe the distribution frequency of samples with the same label (default or non-default) that nearest to ${X_i}$, and then the null hypothesis H0 to be statistical verified is proposed:

H0: For a misclassified sample ${X_i}$, if the distribution frequency of samples with the same label of ${X_i}$ among its nearest $n$ samples satisfy $B\left( {n,p} \right)$ under a specific significance level ${\rm \alpha }$, then ${X_i}$ is a boundary sample.

Accordingly, the alternative hypothesis H1 is: For a misclassified sample ${X_i}$, if the distribution frequency of samples with the same label of ${X_i}$ among its nearest $n$ samples does not satisfy $B\left( {n,p} \right)$ under a specific significance level ${\rm \alpha }$, then ${X_i}$ is a noise sample.

Based on statistical principles, among the nearest $n$ samples ${X_j}\left( {j \in \left\{ {1,2, \cdots ,n} \right\},j \ne i} \right)$ of ${X_i}$, the event that the number of ${X_j}$ that has the same label with ${X_i}$ is 0, marked as $P\left( {{X_i},\; {X_j},n} \right)$, follows the distribution shown in Eq. (2):

(2) $$\matrix{ {P\left( {{X_i},\; {X_j},n} \right) = {{\left( {1 - p} \right)}^n}}. \cr }$$

At a given significance level ${\rm \alpha }$, we need to find the smallest integer $n$ such that:

(3) $$\matrix{ {P\left( {{X_i},\; {X_j},n} \right) \le \alpha}. \cr }$$

Combine Eqs. (2) and (3), taking the logarithm of both sides of the equality sign, we get:

(4) $$\matrix{ {n \times ln\left( {1 - p} \right)\; \le ln\left( \alpha \right)}. \cr }$$

Since the term $ln\left( {1 - p} \right)$ in Eq. (4) is negative, we divide both sides of the inequality sign by $ln\left( {1 - p} \right)$ in Eq. (4) and get:

(5) $$\matrix{ {n \ge \displaystyle{{{\rm ln}\left( {\rm \alpha } \right)} \over {ln\left( {1 - p} \right)}}}. \cr }$$

That is, when observing the nearest $n$ (rounded up to the nearest integer number) samples of a specific sample ${X_i}$, if there is no sample has the same label (default or non-default) of ${X_i}$, then ARPD algorithm can reject the null hypothesis H0 at a significance level of ${\rm \alpha }$ and accept the alternative hypothesis H1, that is, identifies ${X_i}$ as a noise sample. Otherwise, the null hypothesis H0 cannot be rejected at a significance level of ${\rm \alpha }$, then the ARPD algorithm accepts the null hypothesis and identifies ${X_i}$ as a boundary sample. Based on the ARPD algorithm, all samples that cannot be correctly identified by the logistic regression model are screened, and all identified boundary samples are formed into a new dataset ${{\rm D}_b}$, while all identified noise samples are eliminated.

Next, based on ${{\rm D}_b}$, a deep learning model is constructed, the features of the two classes of samples in the boundary area are fully identified. Compared with other credit risk assessment models, the deep learning model can automatically implement feature engineering methods, fully explore the high-dimensional heterogeneous features implicit in the samples in the boundary area, and identify more subtle and complex risk patterns, thereby improving the recognition ability of boundary samples. Considering the computational cost, we build a fully connected neural network with three hidden layers, and the optimal number of neurons and activation function of each hidden layer are determined based on the generalization performance of the deep learning model on the validation set.

In addition, given the apparent “black box” nature of deep learning models, it is difficult for users to clearly understand the relative contribution of each variable to the prediction result. The SHAP algorithm as a representative explainable artificial intelligence (XAI) tool, is further introduced to reveal the impact of each variable in the model on the final prediction result, thus improving the *post-hoc* interpretability. The SHAP algorithm is based on the concept of Shapley value in game theory. It calculates the marginal contribution of each variable to the model output under multiple combinations. The higher the Shapley value of a variable, the more important it is in interpreting credit risk. Equations (6) and (7) respectively show the calculation process of the Shapley value of a given variable $i$ under different feature combinations by the SHAP algorithm, as well as the total Shapley value after summation:

(6) $$\matrix{ {SP\left( {{x^i}} \right) = \mathop \sum \limits_{S \in \left\{ {{x^1},{x^2}, \cdots ,{x^k}} \right\}\backslash \left\{ {{x^i}} \right\}} \displaystyle{{\left| S \right|!\left( {p - \left| S \right| - 1} \right)!} \over {p!}}\left( {val\left( {S \cup \left\{ {{x^i}} \right\}} \right) - val\left( S \right)} \right)} \cr }$$

(7) $$\matrix{ {va{l_i}\left( S \right) = \int \hat f\left( {{x^1},{x^2}, \cdots ,{x^k}} \right)d{P_{x \notin S}} - {E_X}\left( {\hat f\left( X \right)} \right)} \cr }$$

where $S$ represents the set of variables contained in each model, $x$ is the specific sample to be interpreted, $X$ is the vector of all samples, $k$ is the number of variables for each sample, and $val\left( S \right)$ is the prediction result on $S$.

Finally, on the subset ${{\rm D}_l}$ that the logistic regression model can correctly identify samples in it, and on the subset ${{\rm D}_b}$ for building the deep learning model, agglomerative clustering is performed to generate ${\rm N}\left( {\sqrt {{{\rm D}_l}} } \right)$ and ${\rm N}\left( {\sqrt {{{\rm D}_b}} } \right)$ cluster centers respectively as representative samples in different sample space, and function $N\left( \cdot \right)$ measures the round up number of samples in the corresponding dataset. In the application or testing stage, for specific sample ${X_p}$ that need to be identified, the similarity between ${X_p}$ and each cluster center ${C_q}$ is calculated according to Eq. (8), where ${X_p}$ and ${C_q}$ both have $k$ dimensions:

(8) $$\matrix{ {dist\left( {{X_p},\; {C_q}} \right) = \mathop \sum \limits_{a = 1}^k {{\left( {{X_{pa}} - {C_{qa}}} \right)}^2}}. \cr }$$

If the most similar cluster center comes from ${{\rm D}_l}$, the label of ${X_p}$ is predicted by the logistic regression model. If the most similar cluster center comes from ${{\rm D}_b}$, then the label of ${X_p}$ is predicted by the deep learning model.

**Figure 2:**
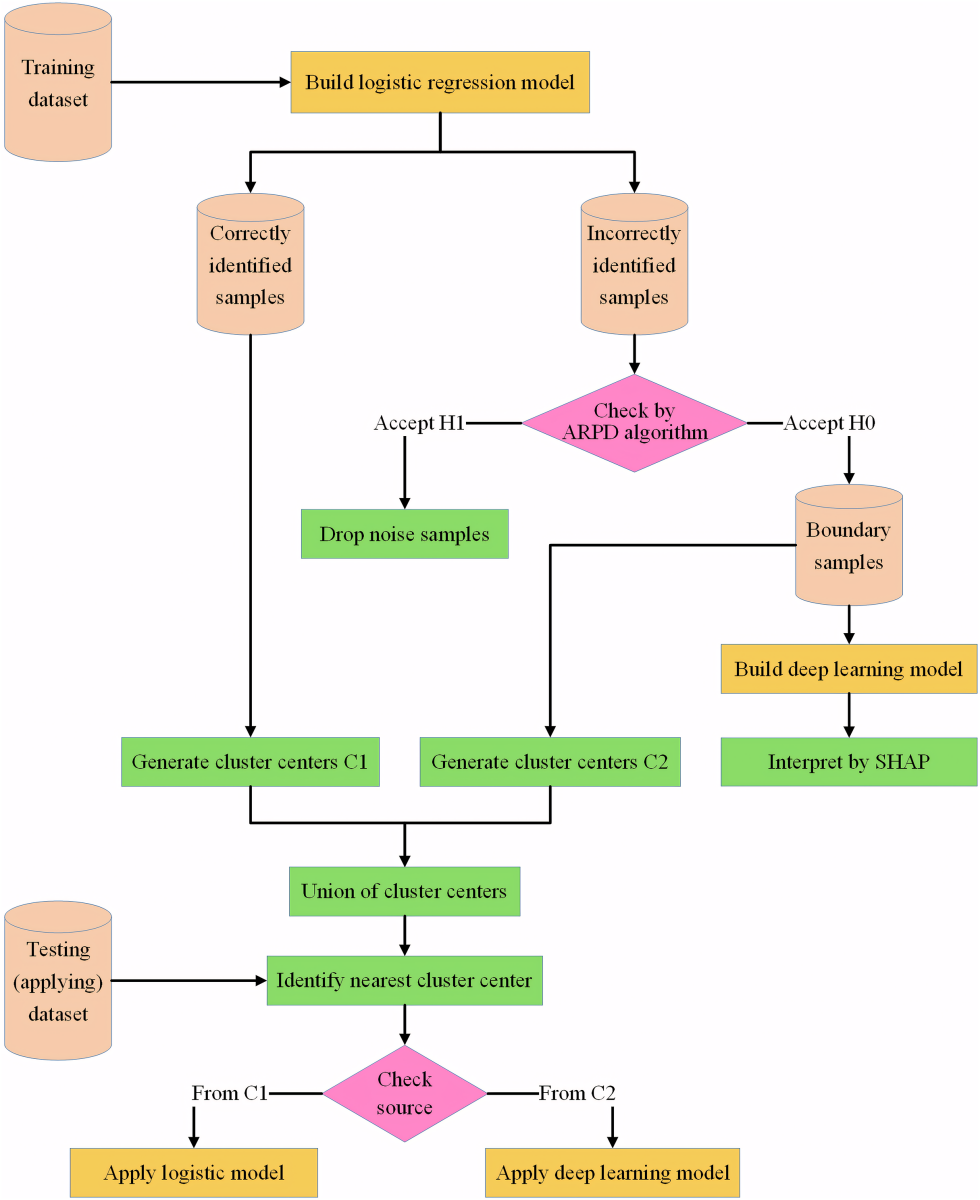
Framework of the proposed IAIBS model.

The framework of the IAIBS model can be summarized as shown in Fig. 2.

With reference to representative literature (Rudin et al., 2022; Runchi, Liguo & Qin, 2023), the overall interpretability of the IAIBS model is analyzed from three aspects: its calculation process, its output, and the relative importance of its variables.

In terms of the interpretability of its calculation process, the IAIBS model first construct a logistic regression model with good self-interpretability, and then naturally divide the whole training dataset into two subsets: correctly identified and incorrectly identified. Then, the proposed ARPD algorithm is used to identify boundary samples and remove noise samples from the incorrectly identified subsets. The relevant calculation processes are mainly based on the transparent statistical methods, so they all have good self-interpretable features. Next, a non-interpretable deep learning model is built on the boundary samples dataset, and the SHAP algorithm is applied to identify the contribution of each variable in the model, which improves the *post-hoc* interpretability. Finally, a self-interpretable agglomerative clustering algorithm is used on the training dataset to extract several cluster centers, and the most appropriate sub-model is selected for predicting risk labels based on the feature similarity between each sample to be evaluated and each cluster center. Overall, most of the calculation processes have obvious self-interpretable features. For deep learning sub-model whose calculation process is difficult to interpret directly, the interpretability is also improved by using the SHAP algorithm.

In terms of the interpretability of the output, the output of both the logistic regression sub-model and the deep learning sub-model are distributed in the interval [0,1], which represents the probability of default for a particular sample, thus is understandable to the users and regulators.

In terms of the interpretability of the relative importance of variables, in the logistic regression model, the positive and negative signs of the regression coefficients represent the direction of each variable’s impact on credit risk, and the absolute value of the standardized regression coefficient also reflects the degree of each variable’s impact on credit risk. In the deep learning sub-model, although the total weights of variables in the neural network are difficult to interpret directly, after being processed by the SHAP algorithm, users can intuitively observe the relative importance of each variable. Therefore, the relative importance of variables can also be interpreted in the IAIBS model.

In summary, the IAIBS model has good interpretability.

The performance of the IAIBS model is further verified through empirical research.

In terms of empirical datasets, four public large-scale datasets commonly used in current research are selected, namely the Personal consumption loan dataset from the datafountain community (abbreviated as PCL dataset, download address: https://www.datafountain.cn/datasets/6218), the credit risk assessment dataset from the FICO website (abbreviated as FICO dataset, download address: https://www.kaggle.com/datasets/averkiyoliabev/home-equity-line-of-creditheloc), the Credit Card Fraud Detection dataset from the Kaggle community (abbreviated as CCF dataset, download address: https://www.kaggle.com/datasets/mlg-ulb/creditcardfraud), and the L&T Vehicle Loan Default Prediction dataset (abbreviated as VL dataset, download address: https://www.kaggle.com/datasets/avikpaul4u/vehicle-loan-default-prediction?select=train.csv). The basic features of the four datasets can be summarized as shown in Table 1, where the imbalance ratios column measures the distribution ratio of non-default samples to default samples in each dataset.

**Table 1:** Basic features of the empirical dataset.

Dataset	Sample size	Total features	Non-default samples	Default samples	Imbalance ratios
PCL	10,000	30	8,317	1,683	4.94
FICO	10,459	23	5,000	5,459	0.92
CCF	284,807	28	284,315	492	577.88
VL	233,154	40	182,543	50,611	3.60679

From Table 1, we can find that the CCF dataset has the largest size with 284,807 samples, followed by the VL and FICO datasets, while the PCL dataset has the smallest size with 10,000 samples. At the same time, the distribution balance of default and non-default samples in the CCF dataset is the weakest, with an imbalance ratio of 577.88, while the distribution of the two classes of samples in the FICO dataset is the most balanced. In addition, the VL dataset has the largest number of variables as 40, while the FICO dataset has the smallest number of variables as 23. In general, experiments on four datasets with different sample sizes, number of variables and imbalance ratios can fully test the performance of the IAIBS model.

In terms of the comparison group models, 11 models widely used in existing studies are selected, namely the logistic regression model (logistic) and the naive Bayes model (GaussianNB) that designed on statistical principles, and machine learning models with a single structure, namely the decision tree model (DT), the support vector machine model (SVM), the deep learning model (DNN) with a fully connected structure, the convolutional neural network model (CNN), and the machine learning models with ensemble or hybrid structures, such as the random forest model (RF), the adaptive boosting model (AdaBoost), the gradient boosting decision tree model (GBDT), the extreme gradient boosting model (XGBoost) and the light gradient boosting machine model (lightGBM).

In terms of model implementation, the XGBoost model is built based on the xgboost package of Python, the lightGBM model is built based on the lightgbm package, the DNN and CNN models are built based on the keras package, and the remaining models are built based on the sklearn package. In the IAIBS model, two sub-models are built based on the sklearn package and the keras packages respectively, and agglomerative clustering is implemented based on the sklearn.cluster package.

In terms of parameter tuning, the hyperopt package of Python is used to tune the hyperparameter combination of each model, and the maximum number of optimization iterations is set to 200 times. In terms of the performance evaluation, seven evaluation indicators, including accuracy, sensitivity, specificity, AUC, gmean, F-score and Matthews correlation coefficient (MCC), are further constructed. The definitions of these indicators refer to Runchi, Liguo & Qin (2023), Xu & Zhang (2025), *etc*. The higher the value of each evaluation indicator, the better the model’s performance.

In terms of experimental design, based on the 5-fold crossover method, the entire dataset is divided into independent training and testing dataset for five times. Each time, the IAIBS model and 11 comparison group models are constructed on the training dataset, and the performance of the above 12 models on the testing dataset is observed. Finally, the average performance of the seven evaluation indicators on the testing dataset after the 5-fold crossover is reported. Besides, considering the sample imbalance problem, in order to ensure the fairness of the comparison, the SMOTE-ENN algorithm (Xiao et al., 2021) is applied to balance the training dataset at each fold, and then 12 models are constructed on the balanced dataset. In addition, in the baseline comparison, the number of cluster centers C1 generated on the samples correctly identified by the logistic regression model, and the number of cluster centers C2 generated on the boundary samples, are set to be the square root of the corresponding dataset size (rounded up). The significance level ${\rm \alpha }$ of the ARPD algorithm is set to be 10%. In the robustness analysis part, the model’s performance under other parameter combinations is verified.

## Results

### Basic comparison results

Table 2 first shows the performance of each model in seven evaluation dimensions on the four experimental datasets, all the numbers are in percentage units (same as below). The best performance of each dimension on each dataset is shown in bold. We can find that on the PCL dataset, the IAIBS model obtains the optimal value under all seven evaluation dimensions, showing a superior generalization ability. Meanwhile, the performance of the ensemble structure models, such as RF, AdaBoost, GBDT, XGBoost, lightGBM, *etc*., is also generally superior to the other models with a single structure.

**Table 2:** Comparison of model performance on four datasets. The best performance of each dimension on each dataset is shown in bold.

Dataset	Model	Accuracy	Sensitivity	Specificity	AUC	gmean	F-score	MCC
PCL	Logistic	74.80	88.19	70.92	86.63	79.04	53.40	46.11
	GaussianNB	74.11	88.00	69.91	83.42	77.84	53.71	46.05
	DT	76.95	88.96	74.35	86.81	80.74	56.79	51.04
	SVM	66.90	74.01	64.30	69.65	69.73	41.56	29.21
	DNN	64.20	68.98	64.30	64.64	63.98	40.19	25.93
	CNN	60.18	82.12	55.31	68.22	65.14	40.37	29.98
	RF	76.05	71.13	78.16	78.36	65.71	44.33	39.86
	AdaBoost	78.32	90.78	74.63	87.67	82.28	58.39	49.90
	GBDT	76.26	92.27	74.03	86.93	83.09	56.63	51.33
	XGBoost	76.15	91.82	74.43	87.22	82.19	57.22	49.73
	lightGBM	76.61	90.05	74.73	88.17	81.92	56.70	49.16
	IAIBS	**79.32**	**93.27**	**79.16**	**89.17**	**84.09**	**59.39**	**52.33**
FICO	Logistic	71.52	77.45	65.09	77.48	70.92	73.93	43.02
	GaussianNB	70.98	73.95	67.75	75.17	70.76	72.67	41.81
	DT	70.27	76.68	63.24	76.00	69.54	72.89	40.45
	SVM	71.78	76.47	66.67	71.57	71.37	73.87	43.41
	DNN	71.79	79.41	63.55	71.48	70.94	74.59	43.70
	CNN	71.74	75.65	67.49	71.57	71.44	73.64	43.33
	RF	72.41	72.31	**72.40**	79.31	72.12	73.14	45.00
	AdaBoost	72.69	74.18	71.04	79.25	72.58	73.92	45.25
	GBDT	72.46	76.44	68.11	79.06	72.00	74.28	44.95
	XGBoost	72.67	75.20	69.75	79.15	72.09	74.05	45.49
	lightGBM	72.30	76.48	67.70	78.27	71.86	74.21	44.52
	IAIBS	**74.55**	**81.69**	70.43	**79.86**	**74.20**	**76.51**	**45.53**
CCF	Logistic	97.45	86.69	98.22	95.55	93.84	86.71	84.56
	GaussianNB	96.12	88.30	96.90	96.33	92.49	80.90	79.35
	DT	93.55	87.90	93.89	96.04	91.93	73.18	71.80
	SVM	97.36	82.04	97.00	91.01	90.52	88.19	84.96
	DNN	97.40	85.30	97.70	92.50	92.20	86.95	84.99
	CNN	96.85	80.63	98.46	89.55	89.04	83.57	82.90
	RF	95.99	89.90	96.59	96.43	93.16	80.76	79.44
	AdaBoost	95.54	87.92	95.89	96.17	93.85	79.20	77.79
	GBDT	97.52	90.54	97.82	96.37	94.28	86.79	85.17
	XGBoost	97.10	90.18	97.82	96.69	94.19	87.50	84.64
	lightGBM	97.12	87.90	97.82	96.41	93.87	85.14	83.99
	IAIBS	**97.56**	**91.73**	**98.73**	**97.48**	**94.74**	**88.69**	**85.42**
VL	Logistic	59.32	61.57	57.06	61.91	59.17	60.18	18.69
	GaussianNB	58.11	68.17	45.16	59.50	56.09	62.83	17.12
	DT	58.93	59.39	58.31	61.28	57.30	58.17	18.37
	SVM	59.73	67.07	52.38	59.73	59.25	62.47	19.68
	DNN	59.49	63.05	55.92	59.48	59.35	60.85	19.03
	CNN	58.52	70.25	46.77	58.51	57.30	62.87	17.53
	RF	61.33	64.10	58.49	64.37	60.86	62.23	**22.82**
	AdaBoost	60.69	60.59	60.74	63.73	60.53	60.55	21.41
	GBDT	60.26	57.63	62.92	63.41	58.84	58.52	21.30
	XGBoost	59.89	67.16	52.55	62.48	59.09	62.43	20.09
	lightGBM	58.34	50.65	62.18	60.59	47.95	49.09	17.06
	IAIBS	**62.70**	**71.51**	**63.97**	**66.03**	**61.55**	**63.31**	19.84

On the FICO dataset, the IAIBS model achieves the best performance in the six evaluation dimensions of accuracy, sensitivity, AUC, gmean, F-score and MCC. Although it did not achieve the highest value in the specificity dimension, it was only 1.97 away from the optimal result of 72.4, which is significantly better than the other models except RF and AdaBoost. At the same time, the sensitivity values of RF and AdaBoost are lower than the other models, indicating that their higher recognition ability for non-default samples comes at the expense of their recognition ability for default samples. Overall, the IAIBS model can better balance the recognition ability of the two classes of samples.

On the CCF dataset, the IAIBS model achieves the best performance in all seven evaluation dimensions. The highest values of accuracy, sensitivity, specificity, AUC, gmean, F -score and MCC are 97.56, 91.73, 98.73, 97.48, 94.74, 88.69 and 85.42 respectively. At the same time, the performance of the 12 models is significantly better than when they are built on the other two datasets. This finding shows that the nature of the dataset is an important factor affecting the performance of most credit risk assessment models.

On the VL dataset, the IAIBS model achieves the best evaluation results in the six evaluation dimensions of accuracy, sensitivity, specificity, AUC, gmean and F-score. Intuitively, the performance advantage of the IAIBS model is more obvious than that of the 11 comparison group models. At the same time, its MCC value is 19.84, which is better than the other seven models in the comparison.

In general, on the four datasets with differentiated features, the IAIBS model showed better evaluation results in most of the evaluation indicators compared to the 11 comparison group models.

### Significance test of performance advantage

Although Table 2 shows that the performance of the IAIBS model is somewhat superior, whether this superiority is statistically significant requires further verification. For this purpose, two non-parametric statistical test methods are applied, namely the Friedman test and the Nemenyi test. The Friedman test evaluates the model performance by ranking the evaluation indicator values of all the models in comparison. If the difference in the sum of the rankings between all the models is large enough, the Friedman test can reject the null hypothesis that all models have the same statistical performance. However, the Friedman test cannot identify which models have significant differences in performance from the rest of the models. After it rejects the null hypothesis, the Nemenyi test further identifies the performance differences between each pair of models by calculating the critical difference value. If the difference between the mean ordinal values of two models exceeds the critical difference value, it is considered that there is a statistically significant difference in the performance of these two models.

**Figure 3:**
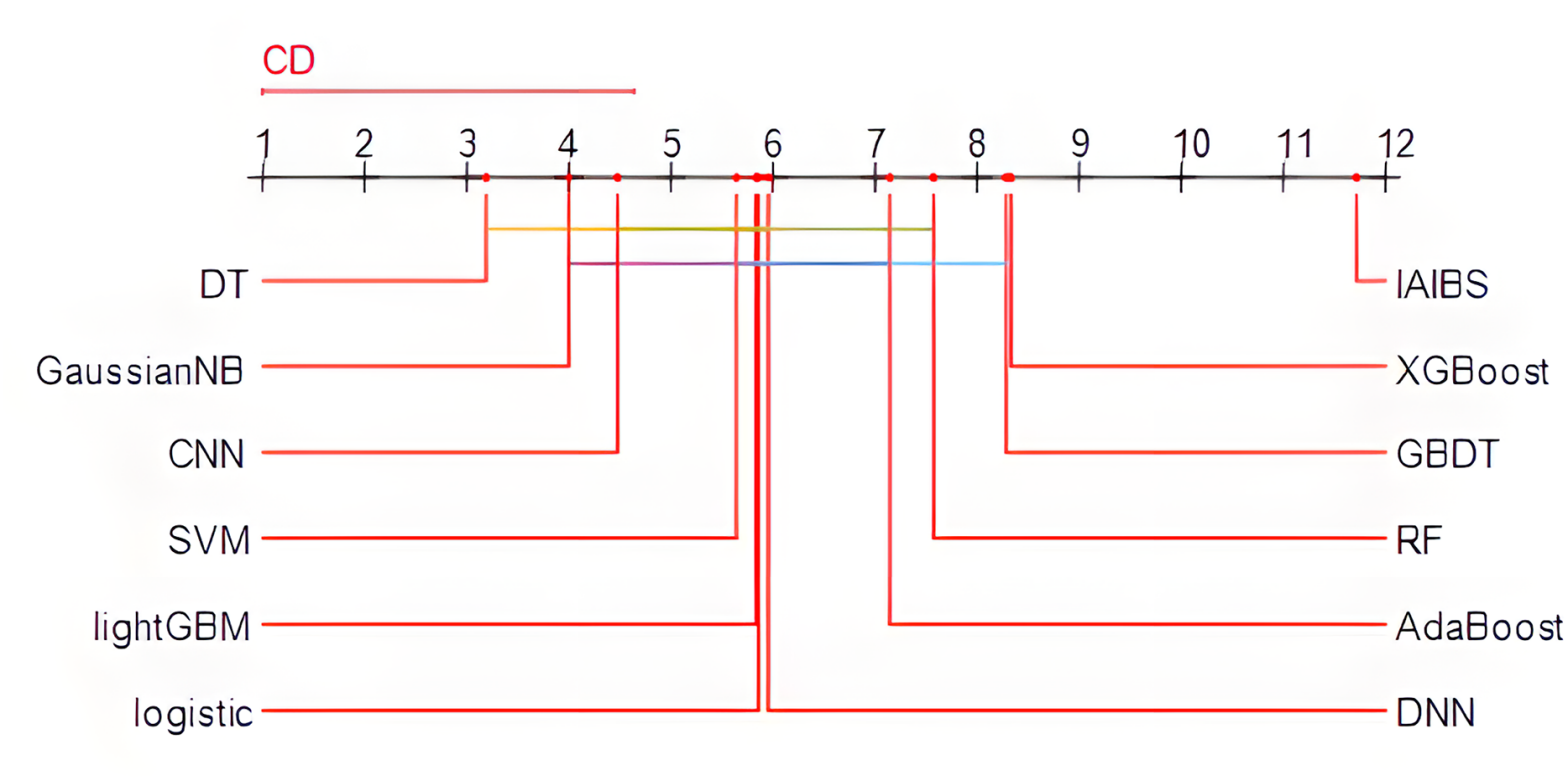
Nemenyi test results.

Specifically, the Friedman test is first applied, the *p*-value of the test result is 2.0853e−15, which is far less than 0.01, indicating that there are obvious performance differences among the 12 models in the comparison. Then, the Nemenyi test is further applied to identify the specific models with obvious performance differences. Figure 3 shows the results of the Nemenyi test with a significance level of 10%. The performance of the models connected by horizontal lines are statistically similar, while the models not connected by any horizontal lines with other models can pass the Nemenyi test at a significance level of at least 10%.

From Fig. 3, we can see that the 11 comparison group models are all connected to at least one other model by a horizontal line, while the IAIBS model is not connected to any other model, indicating that the performance of the IAIBS model is statistically significantly superior to the other 11 comparison group models.

### Robustness analysis under different parameter settings

The performance robustness of the IAIBS model under different hyperparameter settings is further verified.

After training the IAIBS model, it is further necessary to form C1 cluster centers on the dataset that containing samples correctly identified by the logistic regression model, and C2 cluster centers on the dataset containing all the boundary samples. In the absence of prior knowledge about the optimal setting of C1 and C2, the IAIBS model defaults to set them as the square root of the number of samples contained in the two datasets. In the robustness testing stage, we try to change different values of C1 and C2, then observing the performance changes of the IAIBS model.

Specifically, on the four datasets, suppose the dataset correctly identified by the logistic regression model contains $n$ samples and the boundary sample dataset contains $m$ samples, we set the change intervals of C1 and C2 as: $\left[ {0.5 \times \sqrt n ,\; 1.5 \times \sqrt n } \right]$ and $\left[ {0.5 \times \sqrt m ,\; 1.5 \times \sqrt m } \right]$, respectively. Considering the computational cost, the change intervals of C1 and C2 are divided into 100 equal parts (rounded to the nearest integer), and the performance of the IAIBS model is recorded under different combinations of C1 and C2.

**Figure 4:**
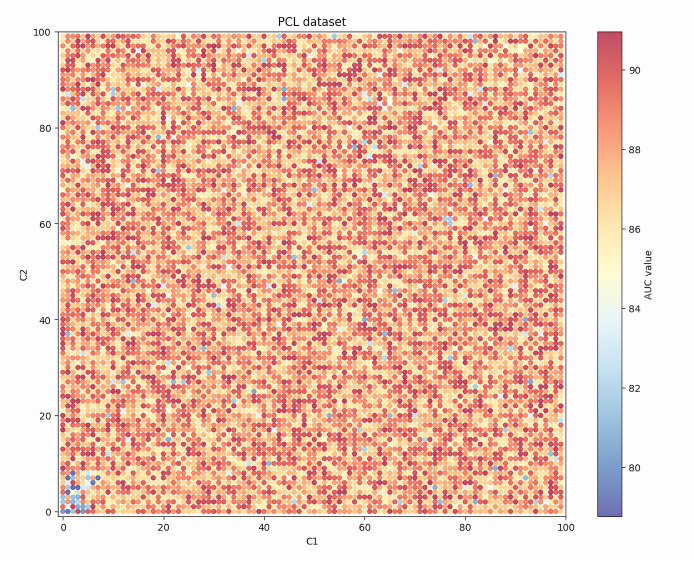
Performance robustness analysis on the PCL dataset.

**Figure 5:**
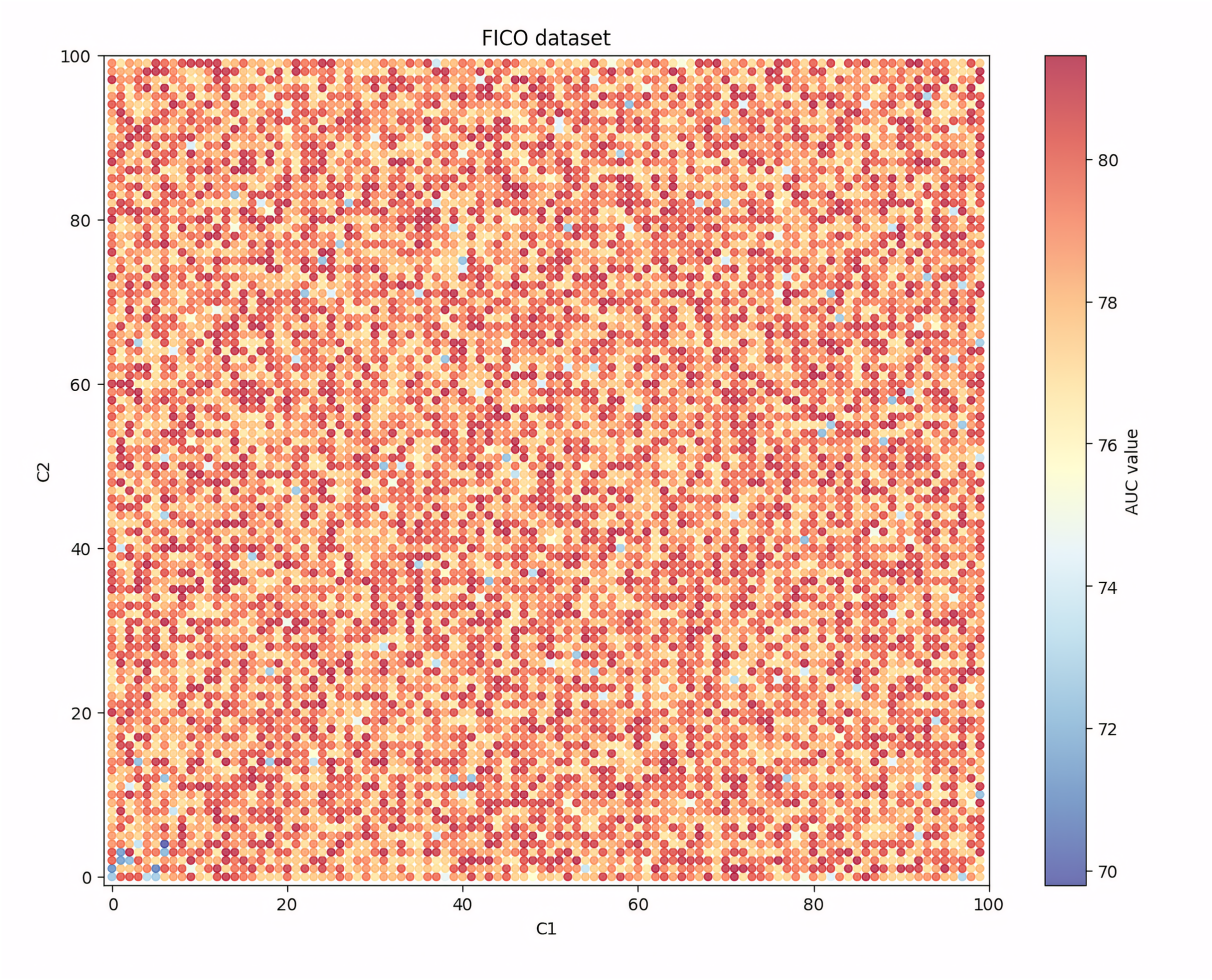
Performance robustness analysis on the FICO dataset.

**Figure 6:**
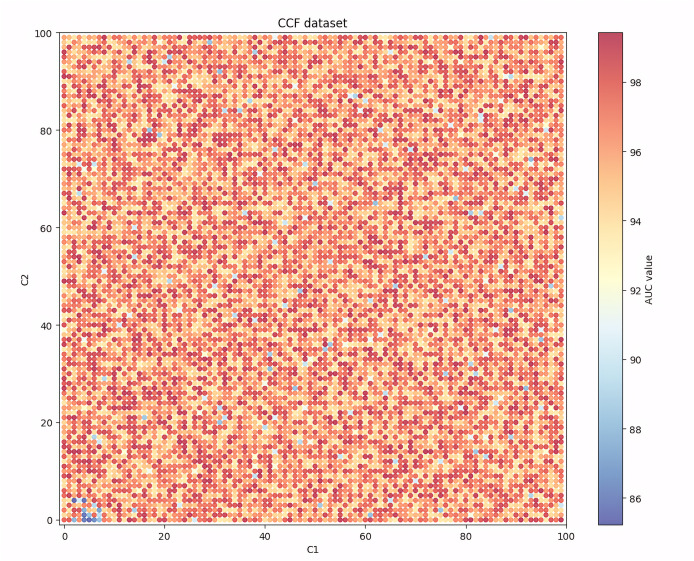
Performance robustness analysis on the CCF dataset.

**Figure 7:**
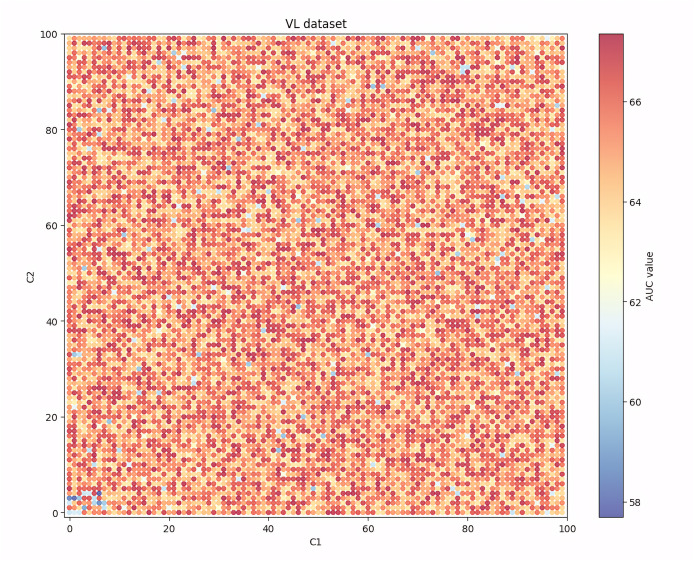
Performance robustness analysis on the VL dataset.

Given that among the seven evaluation indicators, the AUC indicator can show the balanced recognition ability of the model for the two classes of samples, and it is one of the most widely used indicators in the existing research, Figs. 4, 5, 6 and 7 show the changes in the AUC indicator under different combinations of C1 and C2 on the PCL, FICO, CCF and VL dataset, respectively.

Observing Fig. 4, it can be found that on the PCL dataset, when the values of C1 and C2 are small, the generalization ability of the IAIBS model is relatively weak and the AUC values fluctuate greatly. When the values of C1 and C2 are both increased, the AUC continues to increase, but when they are set relatively large, the AUC show a certain downward trend. Overall, the moderate setting of C1 and C2 is conducive to maintaining the IAIBS model with good generalization ability.

From Fig. 5, we can see that in the FICO dataset, when the values of C1 and C2 are both set to be small, the AUC value is relatively low as close to 72. At the same time, when the values of C1 and C2 are both set to be large, the AUC value will also decrease, but the amplitude and frequency of the decrease will be less than when the values of C1 and C2 are both set to be small. At the same time, when the values of C1 and C2 are both set in a moderate range, the AUC value will fluctuate stably around 78.

Further observation of Fig. 6 shows that on the CCF dataset, although the performance of the model will decrease when both C1 and C2 are set to be small or large, the frequency of the decrease is relatively limited, and in most cases the model can achieve a high AUC value that is close to the optimal one. This finding shows that the nature of the dataset is also an important factor affecting the robustness of the model performance. When the variables contain more valuable risk information, the robustness of the IAIBS model is superior.

From Fig. 7, we can see that on the VL dataset, the performance of the model also decreases when the C1 and C2 values are set to be too large or too small. At the same time, the performance degradation of the model is greater when the values are set too small than when they are set too large.

Table 3 shows the maximum (max), minimum (min), mean (mean) and standard deviation (std) of the model’s AUC under different parameter settings on the above four datasets.

**Table 3:** Comparison of model robustness on four datasets.

Dataset	Max	Min	Mean	Std
PCL	90.95	80.19	88.14	1.71
FICO	80.66	72.93	78.02	1.98
CCF	98.45	94.58	98.34	1.63
VL	66.69	60.36	65.30	0.92

From Table 3, we can find that on the four datasets, the AUC indicator under different parameter settings has a mean value closer to the highest value than the lowest value, and the standard deviation is relatively small, indicating that the robustness of the IAIBS model is still maintained under different parameter settings. Moreover, further calculation shows that the coefficients of variation of AUC on the PCL, FICO, CCF and VL datasets are 0.0149, 0.025, 0.017 and 0.014, respectively, which are all low, indicating that the performance fluctuation of the IAIBS model under different parameter settings is relatively limited.

Based on the above results, we can conclude that the performance of the IAIBS model is robust.

### Marginal contribution of each module

The marginal contribution of each key module in the IAIBS model to the overall performance is further analyzed. Specifically, we try the following settings in the IAIBS model respectively:

Setting 1: Replace the deep learning sub-model with a logistic regression sub-model and rebuild the IAIBS model with other settings unchanged, to verify the marginal contribution of the deep learning sub-model.

Setting 2: Replace the logistic regression sub-model with a deep learning sub-model and rebuild the IAIBS model with other settings unchanged, to verify the marginal contribution of the logistic regression sub-model.

Setting 3: Skip the step of removing noise samples with the ARPD algorithm and directly build a deep learning sub-model based on all misclassified samples, to verify the marginal contribution of the ARPD algorithm.

Table 4 shows the performance of the IAIBS model on the four datasets under the above different settings, where “–” represents the performance of the basic IAIBS model, which is consistent with the results as shown in Table 2.

**Table 4:** Performance of the IAIBS model under different settings.

Dataset	Setting	Accuracy	Sensitivity	Specificity	AUC	gmean	F-score	MCC
PCL	–	79.32	93.27	79.16	89.17	84.09	59.39	52.33
	Setting 1	76.31	88.70	76.86	83.11	81.23	56.42	51.39
	Setting 2	76.15	91.40	76.39	85.34	83.00	58.50	49.09
	Setting 3	77.10	89.91	77.10	84.89	82.24	57.79	51.44
FICO	–	74.55	81.69	70.43	79.86	74.20	76.51	45.53
	Setting 1	70.69	73.37	61.97	70.67	70.05	73.04	42.06
	Setting 2	71.86	76.65	66.65	71.65	71.47	73.98	43.56
	Setting 3	72.64	76.89	68.06	79.27	72.27	74.56	45.24
CCF	–	97.56	91.73	98.73	97.48	94.74	88.69	85.42
	Setting 1	93.57	90.75	95.90	94.82	92.23	74.26	73.61
	Setting 2	96.69	90.04	97.33	97.23	93.58	83.00	81.73
	Setting 3	95.45	88.94	96.09	96.39	92.42	78.06	76.61
VL	–	62.70	71.51	63.97	66.03	61.55	63.31	19.84
	Setting 1	59.89	62.42	57.48	63.06	59.45	60.66	20.16
	Setting 2	60.74	67.32	54.08	63.59	60.01	63.01	21.78
	Setting 3	61.08	68.03	54.00	63.99	60.36	63.52	22.38

From Table 4, we find that under the above three settings, except for the MCC indicator on the VL dataset, the performance of the IAIBS model decreases to varying degrees in the remaining evaluation dimensions. Specifically, on the four datasets, when setting 1 is applied, the performance of the model is relatively the weakest in most evaluation dimensions. This shows that when both sub-models are logistic regression models, the IAIBS model’s ability to identify multiple non-linear risk features has declined on large-scale datasets, so it makes sense to apply a deep learning model to fully identify the risk features of samples in the boundary area.

Furthermore, when setting 2 is applied, although the values of each evaluation dimension are reduced to some extent, the degree of reduction is significantly weaker than when setting 1 is applied. This shows that when both sub-models are set as deep learning models, the IAIBS model can better identify the multiple risk features contained in large-scale datasets, but the deep learning model is also prone to overfitting the training samples, so its performance is not as good as when the logistic regression and deep learning models are combined use.

When applying setting 3, the performance is slightly improved compared to setting 2, but it is still weaker than the IAIBS model that includes the ARPD algorithm to remove noise samples under most evaluation indicators. This result shows that the setting of further eliminating misclassified samples and then training a deep learning sub-model based on boundary samples, can effectively remove some noise samples and improve the overall risk feature recognition ability of the IAIBS model.

We also measure the performance volatility in each evaluation dimension under the above three settings (*i.e*., the ratio of the average change in performance under the different settings to the original model performance), Table 5 reports the performance volatility of all the evaluation indicators in the “average” column.

**Table 5:** Performance fluctuation features of the model under different settings.

Dataset	Setting	Accuracy	Sensitivity	Specificity	AUC	gmean	F-score	MCC	Average
PCL	Setting 1	−3.79%	−4.90%	−2.91%	−6.80%	−3.40%	−5.00%	−1.80%	−3.79%
	Setting 2	−4.00%	−2.00%	−3.50%	−4.30%	−1.30%	−1.50%	−6.19%	−4.00%
	Setting 3	−2.80%	−3.60%	−2.60%	−4.80%	−2.20%	−2.69%	−1.70%	−2.80%
FICO	Setting 1	−5.18%	−10.18%	−12.01%	−11.51%	−5.60%	−4.53%	−7.63%	−8.09%
	Setting 2	−3.61%	−6.17%	−5.36%	−10.29%	−3.68%	−3.31%	−4.33%	−5.25%
	Setting 3	−2.57%	−5.87%	−3.36%	−0.75%	−2.60%	−2.54%	−0.63%	−2.62%
CCF	Setting 1	−4.09%	−1.07%	−2.87%	−2.73%	−2.65%	−16.27%	−13.82%	−6.21%
	Setting 2	−0.90%	−1.84%	−1.43%	−0.25%	−1.23%	−6.41%	−4.32%	−2.34%
	Setting 3	−2.16%	−3.04%	−2.68%	−1.11%	−2.45%	−11.98%	−10.31%	−4.82%
VL	Setting 1	−4.48%	−12.70%	−10.13%	−4.50%	−3.41%	−4.19%	1.63%	−5.40%
	Setting 2	−3.13%	−5.86%	−15.46%	−3.70%	−2.51%	−0.47%	9.77%	−3.05%
	Setting 3	−2.58%	−4.86%	−15.57%	−3.09%	−1.93%	0.33%	12.83%	−2.13%

Table 5 shows that the average performance of the models under the three settings decreased by at least 2.13%. When setting 1, setting 2 and setting 3 are applied, the average performance of the models on the four datasets decreased by 5.87%, 3.66% and 3.09% respectively. Based on this, it can be concluded that in the IAIBS model, the average relative contributions of the three modules of building a deep learning sub-model, building a logistic regression sub-model, and using the ARPD algorithm to eliminate noise samples to the model performance are 5.87%, 3.66% and 3.09%, respectively.

### Interpretability of the model

We further train the IAIBS model based on full samples and then interpret it. To avoid repetition and redundancy, we use the PCL dataset as an example for our analysis. Table 6 first shows the parameter estimation results of the logistic regression sub-model on the PCL dataset. The “significance” column shows the significance level of the t-test of the corresponding regression coefficient, while the “constant” column shows the regression coefficient of the constant term. We find except for variables such as use, post_code, region, del_in_18 month, scoring_low, scoring_high, pub_dero_bankrup, recircle_u, initial_list_status, app_type, title, f1 and f4, all the remaining variables can interpret the credit risk of the samples at least at the 10% significance level. At the same time, the positive and negative signs of each regression coefficient reflect the direction of the impact of each variable on the credit risk. A positive regression coefficient indicates that an increase in the variable will bring greater credit risk, while a negative regression coefficient indicates that an increase in the variable will suppress credit risk.

**Table 6:** Parameter estimation results of logistic regression sub.

Variables	Regression coefficient	Significance	Variables	Regression coefficient	Significance
total_loan	0.000	0.037	pub_dero_bankrup	−0.068	0.518
year_of_loan	0.265	0.000	recircle_b	0.000	0.002
interest	0.061	0.000	recircle_u	0.001	0.340
monthly_payment	0.001	0.026	initial_list_status	0.021	0.751
house_exist	0.207	0.000	app_type	−0.184	0.431
censor_status	0.090	0.030	title	0.000	0.847
use	−0.010	0.453	f0	0.035	0.014
post_code	0.000	0.135	f1	−0.699	0.386
region	−0.001	0.758	f2	−0.015	0.004
debt_loan_ratio	0.016	0.000	f3	−0.013	0.100
del_in_18 month	0.032	0.364	f4	0.003	0.780
scoring_low	−0.001	0.397	early_return	−0.698	0.000
scoring_high	0.000	0.792	early_return_amount	−0.001	0.000
known_outstanding_loan	0.048	0.000	early_return_amount_3mon	−0.001	0.055
known_dero	0.217	0.001	Constant	−3.074	0.000

**Figure 8:**
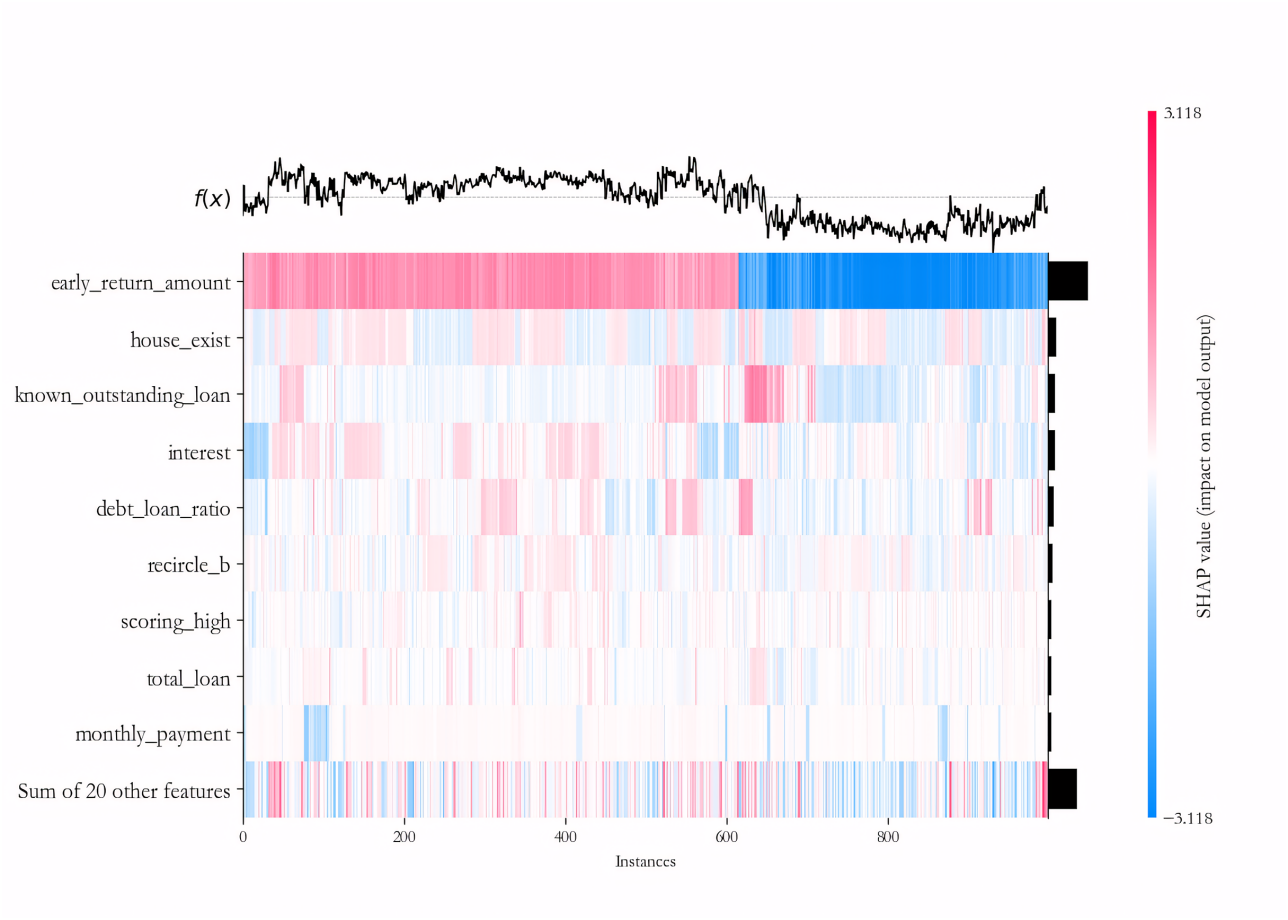
Variable importance analysis on the PCL dataset.

We further explain the importance of each variable in the deep learning sub-model for each sample on the PCL dataset by the SHAP algorithm. To maintain the readability of the final results, 500 samples of default and non-default classes are randomly selected to form the testing dataset, and the IAIBS model is trained on the remaining samples, the predictions are made on the testing dataset and the results are interpreted in Fig. 8, where the horizontal axis represents a particular sample, the vertical axis represents each variable, and the intersection points of the two-dimensional coordinates represent the direction and strength of the influence of each variable on the prediction of credit risk in each sample. The variables with positive explanatory power for credit risk are marked in red, and those with negative explanatory power are marked in blue, with darker colors indicating greater influence.

From Fig. 8 we find that among the testing samples, the early_return_amount variable has the highest explanatory power for the prediction results, and its direction of influence on credit risk is positive in most samples, followed by variables such as house_exist, known_outstanding_loan, and their direction of influence on credit risk is negative in most samples. By comparing Table 6, we can further find that most of the variables that are considered to be more influential by the SHAP algorithm are significant at the 1% significance level in the logistic regression model.

**Figure 9:**
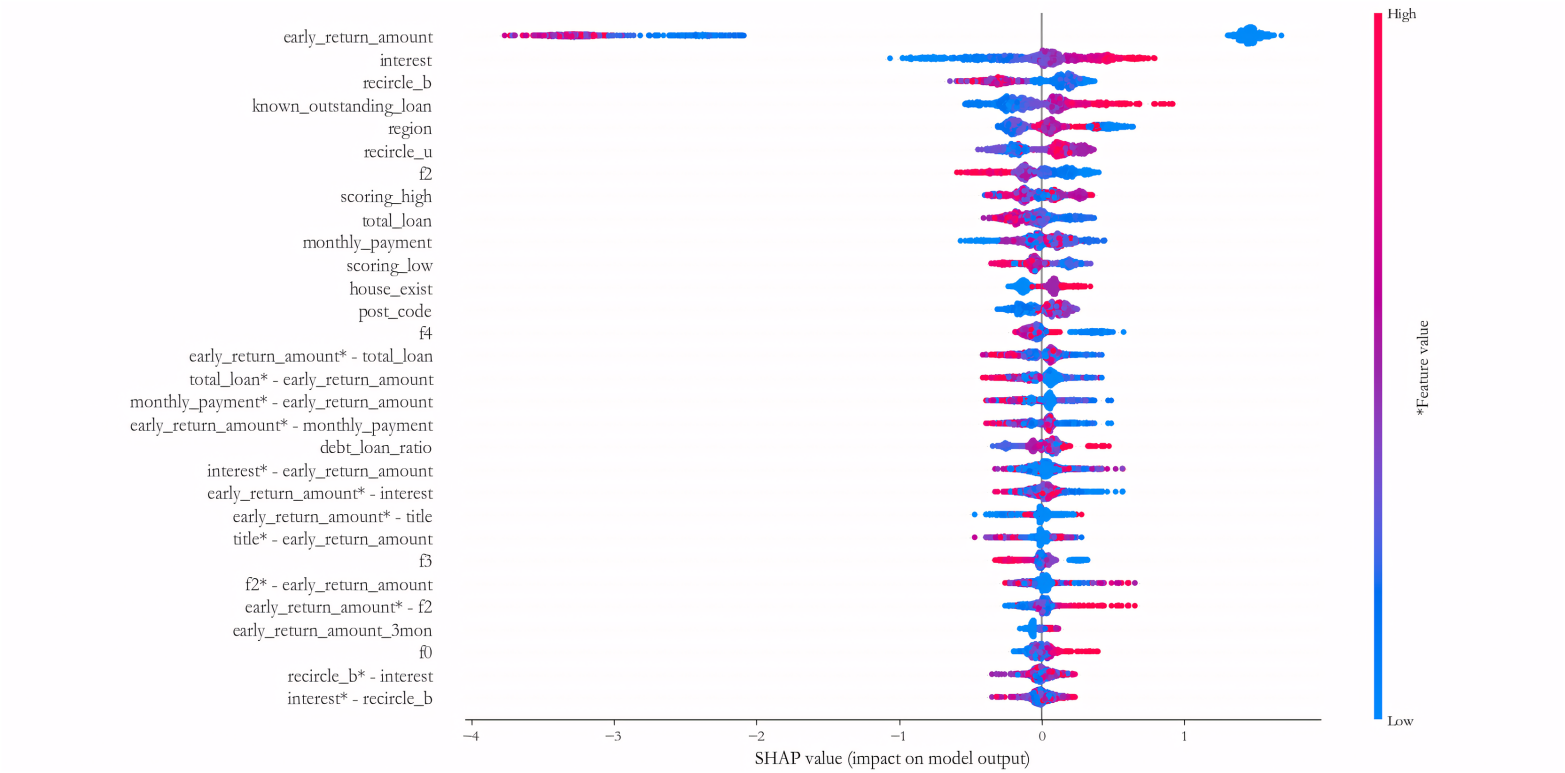
Measuring the 30 variables and key interaction terms.

Figure 9 further shows the results of measuring the 30 variables and key interaction terms that have the maximum impact at the average level on the 1,000 test samples of the PCL dataset. It reveals that in addition to the univariate variables, many interaction variables such as early_return_amount *vs*. total_loan, monthly_payment *vs*. early_return_amount, early_return_amount *vs*. interest, early_ return_amount *vs*. title, f2 *vs*. early_return_amount, recircle_b *vs*. interest, *etc*., also have good explanatory power for credit risk, and the effect is no less influential than most of the univariate variables. This finding suggests that a number of variable combinations have some synergistic influence effects on credit risk, and traditional statistical assessment models are often difficult to measure such effects. Therefore, the construction of DNN sub-model is conducive to identifying such synergistic variable relationships in a more comprehensive way.

**Figure 10:**
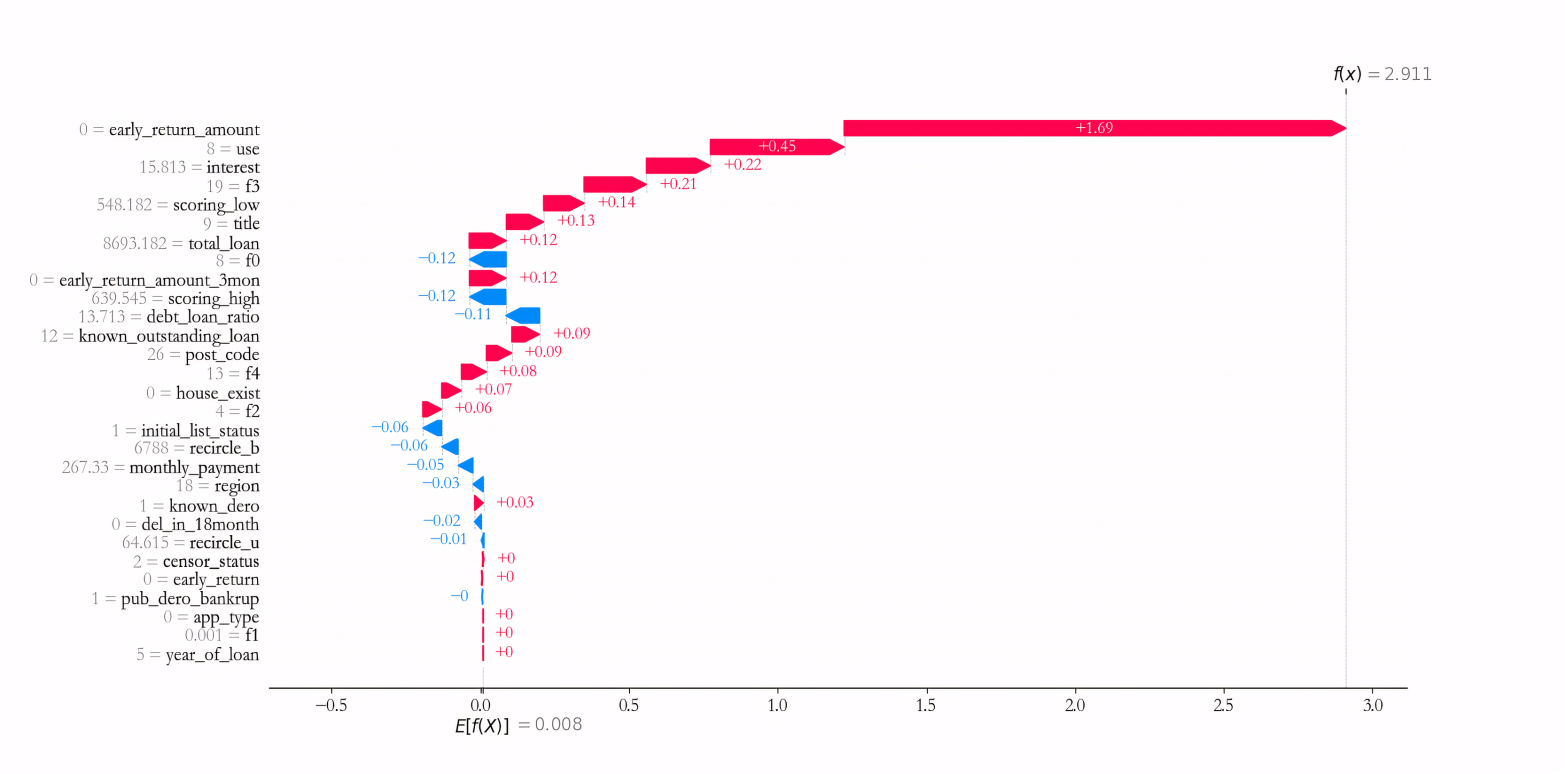
Prediction result of sample 1.

The prediction result of specific sample is further interpreted. Taking sample 1 as an example, as can be seen from Fig. 10, Variables such as early_return_amount, use, interest, f3, scoring_low have the largest positive effect on predicting the credit risk of Sample 1, *i.e*., the larger the corresponding value, the higher the credit risk. Meanwhile, variables such as f0, scoring_high, debt_loan_ratio have the largest negative influence effect on the credit risk of prediction sample 1, *i.e*., the smaller the corresponding value, the smaller the credit risk.

Combined with the results in Table 6, it can be found that the higher the debt size (known_outstanding_loan and debt_loan_ratio), the higher the repayment pressure (total_loan and monthly_payment), the higher the external credit risk score (scoring_high), the higher the credit risk of most of the samples, which is also consistent with the existing credit risk assessment theories (Shay, 1970; Mileris, 2012; Naili & Lahrichi, 2022; Lin et al., 2025), indicating that the IAIBS model can better interpret the formation of credit risk and its prediction mechanism.

Based on the above findings, we can infer that across different credit risk assessment datasets, variables that are meaningful for interpreting general samples tend to have some explanatory power for boundary samples. At the same time, some variables that have low explanatory power for general samples can better explain the risk features of boundary samples. This finding confirms the positive effect of the design of the IAIBS model to construct sub-models according to different sample classes.

## Conclusions

This article proposes a credit risk assessment model that can identify noise samples and boundary samples with interpretable features, namely the IAIBS model. It first fits the training dataset with a logistic regression sub-model with good self-interpretable features. For samples that cannot be correctly identified, the proposed ARPD algorithm is applied to judge whether they are noise samples or boundary samples, and then a deep learning sub-model is further constructed on the boundary samples to fully identify their risk features. Next, the SHAP algorithm is used to perform a *post-hoc* interpretation of the importance of variables in the deep learning model. After the model complete training, several cluster centers are formed by the agglomerative clustering method on the dataset that can be correctly identified by the logistic regression sub-model and the boundary sample dataset. In the application stage, the cluster center closest to the sample to be identified is marked, and the risk label of the sample is predicted by the sub-model constructed on the closest cluster.

Empirical study based on four large-scale datasets shows that compared with the 11 comparison group models, the IAIBS model has better performance in most evaluation indicators, and this performance superiority is supported by the Nemenyi test at a significance level of at least 10%. The robustness test also shows that when the hyperparameter setting is changed, the coefficient of variation of the IAIBS model is small, and the average performance is closer to the optimal value. The marginal contribution analysis further shows that the average relative contribution of the three modules, as building a deep learning sub-model, building a logistic regression sub-model, and using the ARPD algorithm to remove noise samples, to the model’s performance is 6.57%, 3.55% and 3.19%, respectively. Observing the regression coefficient of the logistic regression sub-model and the output of the SHAP algorithm improves the interpretability of the model. At the same time, the interpretation results also show that it is necessary to build several sub-models to identify the risk features of heterogeneous samples.

This study also has some limitations. On the one hand, the ARPD algorithm needs to compute the spatial distance between each misclassified sample and other samples, and when there are many misclassified samples, the time complexity of this computational step is $O\left( {{n^2}} \right)$, which requires more computational resources to be invested when handling large samples. On the other hand, although the SHAP algorithm is applied to interpret the DNN sub-model, this approach still weakens the overall self-interpretable feature of the IAIBS model.

Based on the findings of this study, future research directions worth exploring include identifying and adding more macroeconomic variables that are highly correlated with credit risk, to improve the comprehensiveness of variables for credit risk assessment. Exploring the construction of an ensemble structure model that includes more sub-models with self-interpretable features, to improve the risk identification accuracy while taking into account the interpretable features. Given the fact that real-world credit risk assessment datasets always contain missing values and noisy data as well as unbalanced distributions, novel data cleaning algorithms that can balance interpretability and performance shall be developed to enhance the information value of samples used in the assessment model.

## Supplemental Information

10.7717/peerj-cs.2988/supp-1 Supplemental Information 1 PCL raw data. An online personal loan default prediction dataset based on an anonymous online lender in China, which contains 10,000 samples of 30 dimensions, of which 8,317 are non-defaulted and 1,683 are defaulted. The dataset is available at: https://www.datafountain.cn/datasets/6218. Registration and login are required to download it.

10.7717/peerj-cs.2988/supp-2 Supplemental Information 2 The code of the model in python 3.10.

## Additional Information and Declarations

### Data Availability

The following information was supplied regarding data availability:

The raw measurements are available in the Supplemental Files.

The FICO dataset is available at Kaggle: https://www.kaggle.com/datasets/averkiyoliabev/home-equity-line-of-creditheloc.

The CCF dataset is available at Kaggle: https://www.kaggle.com/datasets/mlg-ulb/creditcardfraud.

The VL dataset is available at Kaggle: https://www.kaggle.com/datasets/avikpaul4u/vehicle-loan-default-prediction.

The code is available in the Supplemental Files.
